# A homemade snare device for removing large foreign bodies

**DOI:** 10.1055/a-2155-6398

**Published:** 2023-09-15

**Authors:** Bo Wu, Qun Zhu, Xincheng Xie, Fulong Zhang, Chunhua Zhou, Yuandong Zhu

**Affiliations:** Department of Gastroenterology, Affiliated Hangzhou Xixi Hospital of Zhejiang University School of Medicine, Hangzhou, China

A 20-year-old man accidentally inserted a rectal massager completely into the rectum and was unable to remove it himself, so he came to our hospital. Abdominal computed tomography scan revealed a rectal foreign body obstructing the intestinal tract ( Fig. 1 ). Emergency surgery attempted to retrieve the item using forceps, but this ultimately failed due to the inability to pass the gap between the foreign body and the intestine.

**Fig. 1:**
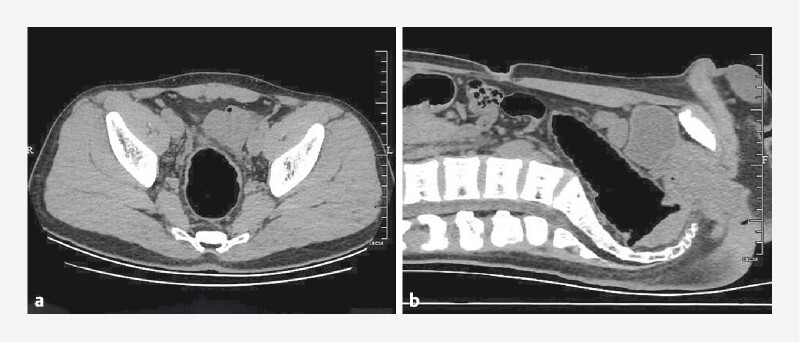
Abdominal computed tomography. **a** The image revealed a rectal foreign body with a density similar to that of the surrounding soft tissues. **b** It was lodged at the junction of the rectum and sigmoid colon, causing intestinal obstruction.

In order to capture and secure the large and irregular foreign body under endoscopy, we constructed a homemade foreign body snare device. Initially, we inserted the ends of a guidewire (AG-5041-3545; AGS MedTech, Hangzhou, China) in reverse through the distal end of a pusher for biliary drainage catheters (BPDS-41993-0709/22; Micro-Tech (Nanjing) Co., Ltd, Nanjing, China), creating an O-shaped snare at the tip of the pusher. The size and tightness of the snare at the tip could be adjusted by manipulating the guidewire. Due to the thinness and inherent tension of the guidewire, it could easily pass through the gap between the foreign body and the rectal mucosa, thereby securing the foreign body in place ( Video 1 ).

**Video 1** Description and use of the foreign body snare device.


Compared with other foreign body retrieval devices 1 2 3 , the snare device has the following advantages:

1. larger opening diameter, capable of snaring large foreign bodies
2. smooth and slender guidewire, which easily passes through the narrow gap between the foreign body and the rectal mucosa
3. easy availability of materials and simple fabrication
4. no concerns about the snare device becoming embedded in and inseparable from the foreign body.

Using this tool, we successfully removed the foreign body ( Fig. 2 ). The patient reported feeling well and was discharged on the same day.

**Fig. 2:**
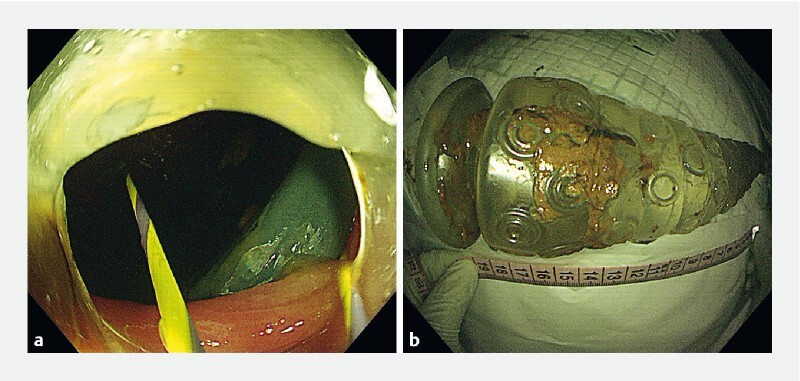
Retrieval of the foreign body. **a** The foreign body was captured and secured using a snare device under colonoscopy guidance. **b** The foreign body measured 19 × 5 × 5 cm in vitro.

Endoscopy_UCTN_Code_TTT_1AQ_2AH

## Notes

Endoscopy E-Videos

https://eref.thieme.de/e-videos
*E-Videos* is an open access online section of the journal *Endoscopy* , reporting on interesting cases and new techniques in gastroenterological endoscopy. All papers include a high-quality video and are published with a Creative Commons CC-BY license. Endoscopy E-Videos qualify for HINARI discounts and waivers and eligibility is automatically checked during the submission process. We grant 100% waivers to articles whose corresponding authors are based in Group A countries and 50% waivers to those who are based in Group B countries as classified by Research4Life (see: https://www.research4life.org/access/eligibility/ ). This section has its own submission website at https://mc.manuscriptcentral.com/e-videos
